# Control of Fungal Diseases and Fruit Yield Improvement of Strawberry Using *Bacillus velezensis* CE 100

**DOI:** 10.3390/microorganisms10020365

**Published:** 2022-02-04

**Authors:** Sarah Hong, Tae Yoon Kim, Sang-Jae Won, Jae-Hyun Moon, Henry B. Ajuna, Kil Yong Kim, Young Sang Ahn

**Affiliations:** 1 Department of Chemical Engineering, University of California, Davis, CA 95616, USA; sarahhong20@gmail.com; 2 Department of Plant Sciences, University of California, Davis, CA 95616, USA; treee757@gmail.com; 3 Department of Forest Resources, Chonnam National University, Gwangju 61186, Korea; lazyno@naver.com (S.-J.W.); mjh132577@naver.com (J.-H.M.); ajunahenry@mmu.ac.ug (H.B.A.); 4 Division of Agricultural and Biological Chemistry, Institute of Environmentally Friendly Agriculture, Chonnam National University, Gwangju 61186, Korea; kimkil@jnu.ac.kr

**Keywords:** biocontrol agent, lytic enzymes, phytopathogenic fungi, auxin, strawberry production

## Abstract

Due to the increasing health and environmental risks associated with the use of fungicides in agriculture, alternatives—such as using plant growth-promoting bacteria (PGPB) to suppress phytopathogens—that simultaneously improve plant yield, are important. This study evaluated the biocontrol efficiency of *Bacillus velezensis* CE100 against *Macrophomina phaseolina* and *Fusarium oxysporum f. sp. fragariae,* the respective causal agents for charcoal rot and fusarium wilt diseases in strawberry, and its potential to enhance strawberry growth and fruit production. *B. velezensis* CE 100 produced fungal cell wall-degrading enzymes, chitinases, and β-1,3-glucanases; and inhibited the mycelial growth of *M. phaseolina* and *F. oxysporum* f. sp. *fragariae* by 64.7% and 55.2%, respectively. The mycelia of both phytopathogenic fungi showed severe swelling and rupturing of the hyphae compared to the smooth, normal growth in the control group. Moreover, *B. velezensis* CE100 produced up to 2.8 units/mL of indole-3-acetic acid (IAA) during incubation and enhanced root biomass in strawberries. Consequently, *B. velezensis* CE 100 not only increased the fruit yield of strawberries by controlling the fungal diseases but also through enhancing plant growth. The findings of this study indicate that *B. velezensis* CE100 could be a safe, ecofriendly biocontrol alternative to chemical fungicides in strawberry production.

## 1. Introduction

Strawberry (*Fragaria × ananassa* Duch.) is an important commercial fruit crop with rich organoleptic qualities such as a pleasant flavor, texture, and taste, and numerous health benefits [1,2]. Strawberries are widely consumed as fresh fruit or processed into various food products [1,2]. Based on the most recent statistics, global strawberry production has increased by 41% in the last decade; and China, the United States of America (USA), the European Union (EU), Mexico, Turkey, Egypt, and South Korea are the leading producers [1,3]. In 2019, the annual strawberry yields in the USA and China were 1,021,490 t from 18,130 ha (563,425 hg/ha) and 3,221,557 t from 126,126 ha (255,424 hg/ha), respectively [1,3]. The increase in strawberry productivity over the past decade could be attributed to scientific innovations in the areas of breeding, improving cultural practices, and pest and disease management [4,5,6].

However, strawberry production is heavily prone to field and post-harvest losses due to high susceptibility to fungal diseases such as root rot, crown rot, charcoal rot, and fusarium wilt disease [7,8,9,10]. Some of the most notable soil-borne fungal pathogens, such as *Macrophomina phaseolina* and *Fusarium* *oxysporum* f. sp. *Fragariae*, have great longevity and exhibit highly competitive saprophytic ability [10,11]. Specifically, *M. phaseolina* is a cosmopolitan fungal pathogen with a wide host range of approximately 500 plant species [12]. It causes crown and charcoal rot diseases which are characterized by the browning of the root’s vascular and cortical tissues and can lead to tremendous losses in strawberry production [12,13]. *M. phaseolina* produces microsclerotia, which are highly resilient and resistant structures that are heat tolerant, making them extremely difficult to control [14]. The microsclerotia enable prolonged survival and overwintering of *M. phaseolina* and are usually the main form of inoculum in subsequent infections [10,14]. In addition, *F. oxysporum* is also a notorious soil-borne pathogen that penetrates strawberry plants through the root system and causes root rot and vascular discoloration of the crown tissues [11,15]. It causes rapid wilting of strawberry plants and reduced yields. It can lead to devastating losses, especially when exacerbated by heat and water stress [8].

To reduce losses caused by phytopathogenic fungal diseases in strawberry production, farmers used to rely on methyl bromide (MB), a soil fumigant [7,10,11]. However, MB soil fumigants are currently restricted in many countries due to their destructive nature to the ozone layer [7,16,17]. Several alternatives to MB, such as iodomethane, methyl iodide, chloropicrin, 1,3-dichloropropene, 1,3-D-methyl isothiocyanate, and metam-sodium/potassium are being used in various jurisdictions [18,19,20]. Soil fumigants such as chloropicrin, and chloropicrin in combination with iodomethane, have demonstrated the ability to protect strawberries from pests and pathogens and can increase yield by 100% [18]. Even so, exposure to soil fumigants, mainly by inhalation, causes toxicity to humans and animals, such as the disruption of fetal growth [16,17,20]. Moreover, excessive application of chemical fungicides causes constant exposure of the phytopathogens to sub-lethal levels of the fungicide, which could potentially lead to the development of fungicide resistance [21]. In addition, quality assurance standards regarding consumer safety from the risk of chronic illness due to the persistence of chemicals in the food, and the environmental protection concerns regarding the effects of chemical fungicides on non-target organisms, are increasingly driving consumers’ preferences, especially in developed countries [22,23,24]. Certainly, future trends in consumers’ preferences will increasingly favor organic over conventionally produced foods, and the premium prices for organic products will gradually cause a shift in food production methods.

Biological control alternatives such as the use of plant growth-promoting bacteria (PGPB) with antagonistic activity against phytopathogenic fungi have been widely reported [25,26,27,28]. Among the PGPB, *Bacillus* spp. are among the most important biocontrol agents that have been widely exploited in the control of plant fungal pathogens [27,28,29,30]. PGPB such as *Bacillus velezensis* control plant diseases by direct competition and inhibition of phytopathogens using antibiotic compounds such as cyclic tetrapeptides, bacteriocins such as colicin-like bacteriocins, and hydrolytic enzymes such as chitinases and β-1,3-glucanases [26,27,28,29,31]. However, a previous study indicated that antifungal compounds obtained from *B. velezensis* (cyclic tetrapeptides) were produced in very low concentrations that cannot effectively control fungal pathogens under field conditions [26]. The fungal and oomycete cell walls which are mainly composed of chitin and β-glucan polymers play a vital role in their survival, multiplication, and host infection [32,33]. Thus, the cell wall could be a suitable target site for the biomolecules used in the control of phytopathogenic fungi and oomycetes [34,35,36]. Thus, cell wall-degrading enzymes produced by PGPB, such as chitinases and β-1,3-glucanases, are valuable alternatives for the biocontrol of plant diseases caused by phytopathogenic fungi and oomycetes [27,29,36].

In addition, PGPB can also enhance plant growth through the secretion of plant growth hormones such as indole acetic acid (IAA) auxin, which enhances root hair growth and lateral root development in plants [27,29,37,38,39]. The improved root architecture increases the rate of nutrient uptake from the soil, which leads to enhanced plant growth and fruit yield [39]. The use of PGPB such as *B. velezensis* CE 100 has also been reported to enhance the growth of forest seedlings mainly through the production of IAA, and increasing nitrogen and phosphorus availability in the soil through ammonium production and phosphate solubilization activity, respectively [27,29,40]. Previous studies have highlighted the multiple functional properties of *Bacillus* spp., including plant disease management and growth promotion [27,28,29,30]. *Bacillus* spp. have previously demonstrated biocontrol potential against various phytopathogens that cause diseases of economic importance in strawberries [6,9,41]. These include *M. phaseolina* which causes charcoal rot, *F. oxysporum f. sp. fragariae* that cause fusarium wilt, and *Botrytis cinerea*, which causes grey mold in strawberries [6,9,41,42]. The biocontrol and bio-stimulation potential of *Bacillus* spp. are attributed to their functional properties, such as the production of antibiotics, lytic enzymes, IAA, ammonia-N, and phosphate solubilization [29,41,42].

However, the potential for simultaneous biocontrol and bio-stimulation potential of PGPB is still a subject of research. Due to the increase in demand for safe, chemical-free food production systems, new research innovations about the use of PGPB to control plant disease and to enhance crop yield are important. Therefore, this study aimed to examine the potential use of *B. velezensis* CE 100 as a biocontrol agent against fungal pathogens of the strawberry, *M. phaseolina,* and *F. oxysporum* f. sp. *fragariae,* while simultaneously enhancing plant growth and fruit yields.

## 2. Materials and Methods

### 2.1. Growth of Bacillus velezensis CE 100

The bacterial strain *B. velezensis* CE 100 used in this study was obtained from Purne Inc. (Jangseong, Korea). *Bacillus velezensis* CE 100 was originally isolated from tomato cultivated soil in Korea as previously described [43]. To examine the growth pattern of *B. velezensis* CE 100, the bacterial strain was obtained from Purne Inc. (Purne Inc., Jangseong, Korea) and inoculated in 2 g/L of pink broth (PB) medium (Purne Inc., Jangseong, Korea) [29]. The medium was supplemented with 3 g/L sucrose (Fisher Scientific, Fair Lawn, NJ, USA) and 3 g/L chemical fertilizer (N:P:K = 20:20:20, Integrated Agribusiness Professionals, Fresno, CA, USA), and incubated at 50 °C in a shaking incubator at 130 rpm for 10 days. During the incubation period, 1 mL of the bacterial culture was sampled daily to enumerate the colony-forming unit (CFUs) using the standard plate count method on nutrient agar (NA) media. In addition, 1 mL of the bacterial culture was sampled daily and used for the analysis of lytic enzyme activity produced by *B. velezensis* CE 100. The experiment was conducted with three replications.

### 2.2. Production of Chitinase and β-1,3-Glucanase by Bacillus velezensis CE 100

For the chitinase and β-1,3-glucanase assays, 1 mL bacterial cultures sampled daily during the incubation period (as indicated above) were centrifuged at 10,000 rpm for 15 min. The supernatants were used for the analysis of the lytic enzyme activity of chitinase and β-1,3-glucanase.

Chitinase activity was determined by estimating the amount of reducing sugars produced per hour, from the hydrolysis of chitin by the enzymatic solution using a modified method described by Moon et al. [44]. Briefly, 50 µL of the enzymatic solution (bacterial supernatant) was mixed with a 500 µL substrate of 0.5% colloidal chitin. The mixture was then mixed with 450 µL of 50 mM sodium acetate buffer (pH 5.0). The contents were incubated at 37 °C for 1 h using Eppendorf tubes submerged in a water bath (Temperature-Controlled Bater Bath 1660504EDU, Bio-Rad Laboratories, inc., Inc., Hercules, CA, USA). The reaction was terminated by adding 200 µL of 1 N NaOH followed by centrifugation at 10,000 rpm for 10 min at 4 °C. The concentration of reducing sugars was estimated calorimetrically based on the loss of color after reacting 750 µL of the solution with 1 mL of Schales’ reagent (0.5 M sodium carbonate and 0.5 g/L potassium ferricyanide in water). The solution was diluted with250 µL of deionized distilled water and boiled at 100 °C for 15 min. Then the optic density was measured at 420 nm (OD_420_) using a UV spectrophotometer (UV-1650PC, Shimadzu, Kyoto, Japan). One unit of chitinase activity was defined as the reducing activity that released 1 µmol of N-acetyl-glucosamine per hour at 37 °C, which is equivalent to a decline of approximately 0.1287 OD_420_.

The activity of the β-1,3-glucanase enzyme was determined by estimating the amount of glucose produced per hour from the hydrolysis of laminarin using a modified method described by Choub et al. [29]. The reaction mixture contained 50 μL of enzyme solution (bacterial supernatant), 50 μL of 1% laminarin solution, and 400 μL of 50 mM sodium acetate buffer (pH 5.0). The solution was incubated at 37 °C for 1 h and the reaction was terminated with 1.5 mL of 3,5-dinitrosalicylic acid (DNS) reagent and boiled in a water bath for 5 min. The increase in optical density at 550 nm was then measured using a UV spectrophotometer (UV-1650PC, Shimadzu, Kyoto, Japan) to determine the concentration of reducing sugars produced. One unit of β-1,3-glucanase activity was defined as the amount of enzyme that catalyzed the release of 1 µmol of glucose per hour at 37 °C, which is equivalent to an increase of approximately 0.7354 OD_550_.

### 2.3. Antagonistic Activity of Bacillus velezensis CE 100 against Fungal Pathogens

The phytopathogenic fungi *M. phaseolina* isolate GL1310 and *F. oxysporum* f. sp. *fragariae* isolate GL1080 [45] were provided by T.R. Gordon Laboratory from the Department of Plant Pathology, University of California, Davis. The phytopathogenic fungi were sub-cultured at 25 °C for seven days using potato dextrose agar (PDA), obtained from Difco Laboratories Inc. (Detroit, MI, USA). The antagonistic activity of *B. velezensis* CE 100 against *M. phaseolina* and *F. oxysporum* f. sp. *fragariae* was examined on PDA plates, using the dual culture method. Briefly, a 6-mm plug of each phytopathogenic fungus from the 7-day-old culture was placed on the left-hand side of the PDA plate using a sterile cork-borer. Then a loop of two-day bacterial culture was streaked at a distance of 4 cm from the phytopathogenic fungal plug, on the right-hand side of the same plate. The control group was prepared by inoculating the phytopathogenic fungi on the PDA plates without *B. velezensis* CE 100. Nine replicates of each group were incubated at 25 °C for 7 days for *M. phaseolina* and 11 days for *F. oxysporum* f. sp. *fragariae*. The inhibition of fungal growth by *B. velezensis* CE 100 was determined using the following formula: inhibition (%) = ((X − Y)/X) × 100, where X represents the radial growth of the phytopathogenic fungi in the control group and Y is the radial growth of the phytopathogenic fungi in the dual culture group [46]. Then small pieces of the phytopathogenic fungal mycelia were gently picked from the fungal growth border in both dual culture and control groups to examine the morphological alterations of the hyphae. The fungal specimens were wet-mounted on glass slides, covered with glass coverslips, and the morphological deformations of *M. phaseolina* and *F. oxysporum* f. sp. *fragariae* were examined at a magnification of 200× using a light microscope (BX41, Olympus, Tokyo, Japan). The mycelial specimens obtained from the dual culture plates were picked from the fungal growth border next to the bacterial streak. All experiments for morphological observation of the mycelia were performed in triplicate.

### 2.4. Quantitative Analysis of Indole-3-Acetic Acid (IAA) Produced by Bacillus velezensis CE 100

The concentration of IAA produced by *B. velezensis* CE 100 was determined calorimetrically based on its oxidation reaction by peroxidase. The amount of IAA oxidized was estimated from the intensity of pink coloration develop by reacting the samples with Salkowski’s reagent, using a modified method described by Choub et al. [29]. The bacterial culture was prepared in a sterilized PB medium amended with 0.6 g/L of yeast extract and 0.1 g/L of L-tryptophan and incubated for 10 days in an H1012 Incu-Shaker set at 30 °C and 140 rpm. For the control, no bacterial inoculation was done. During the incubation, 2 mL of broth cultures were collected daily and centrifuged at 10,000 rpm for 20 min. Then 1 mL of each supernatant was transferred into a 5 mL test tube and mixed with 2 mL of Salkowski’s reagent followed by two drops of phosphoric acid. Salkowski’s reagent was prepared on the same day (about 15 min before use) by adding 1 mL of 0.5 M FeCl_3_ (freshly prepared in deionized water) into 50 mL of 35% perchloride acid at room temperature. The solutions were then incubated at room temperature, under dark conditions for 25 min, and the absorbance was immediately measured at 530 nm using a UV spectrophotometer (UV-1650PC, Shimadzu, Kyoto, Japan). IAA production was calculated based on the increase in the intensity of the pink coloration using an equation obtained from the calibration curve for indole-3-acetic acid standard (Sigma-Aldrich^®^, Darmstadt, Germany).

### 2.5. Growth of Strawberry Seedlings under Greenhouse Experimental Conditions

Albion and Sequoia strawberry seedlings were obtained from Lemuria nursery located in Dixon, CA, USA, in April 2020 and used for the greenhouse experiment. To determine their susceptibility to phytopathogenic fungi, a seedling from each cultivar was pre-infected with either *M. phaseolina* or *F. oxysporum* f. sp. *fragariae*. The disease symptoms were more severe in Albion cultivars infected with *M. phaseolina* and Sequoia cultivars infected with *F. oxysporum* f. sp. *fragariae*. On the other hand, both Albion cultivars infected with *F. oxysporum* f. sp. *fragariae* and Sequoia cultivars infected with *M. phaseolina* only exhibited mild symptoms. Consequently, Albion cultivars were used for *M. phaseolina* infection, and Sequoia cultivars were used for *F. oxysporum* f. sp. *fragariae* infection; both experiments were conducted concurrently. Each strawberry plant was transferred into a 1 L pot filled with premixed soil, and the experiment was performed in a greenhouse located at the University of California (UC), Davis, CA, USA. The greenhouse temperature was maintained in the range of 20 to 25 °C. Plants were grown under natural light and the water was supplied daily by overhead irrigation.

For each experimental group (*M. phaseolina* or *F. oxysporum f. sp. fragariae* infection), three treatments were applied: (i) non-inoculated control (without phytopathogenic fungi or bacteria), (ii) inoculation with only *M. phaseolina* or *F. oxysporum f. sp. fragariae*, and (iii) co-inoculation of *B. velezensis* CE 100 and either *M. phaseolina* or *F. oxysporum* f. sp. *fragariae*. The seedlings were transplanted at the end of April 2020. All plants were maintained under similar greenhouse experimental conditions for the first two weeks before treatment application.

The phytopathogenic fungi *M. phaseolina* and *F. oxysporum* f. sp. *fragariae* were separately cultured on PDA plates amended with 0.01% tetracycline hydrochloride (Fisher Scientific, Fair Lawn, NJ, USA) (PDA-t). Both *M. phaseolina* and *F. oxysporum* f. sp. *fragariae* plates were incubated at 25 °C for five and seven days, respectively. For phytopathogenic fungal infection, two separate inoculations were done in the rhizosphere of each seedling on 4 May and 11 May 2020, respectively. For the first inoculation, two 6-mm plugs of *M. phaseolina* or *F. oxysporum* f. sp. *fragariae* were buried 5 cm under the soil adjacent to the seedlings in the respective treatment groups. When the color of the leaves did not change, then four 6-mm plugs of the phytopathogenic fungi were re-inoculated after a week. For the non-inoculated control group, two 6-mm plugs followed by four 6-mm plugs of PDA-t without phytopathogenic fungi were buried 5 cm in the rhizosphere of the seedlings on 4 and 11 May 2020, respectively.

The bacterial strain was inoculated in 2 g/L of pink broth (PB) medium (Purne Inc., Jangseong, Korea). The media was supplemented with 3 g/L sucrose (Fisher Scientific, Fair Lawn, NJ, USA), 3 g/L chemical fertilizer (N:P:K = 20:20:20, Integrated Agribusiness Professionals, Fresno, CA, USA), and incubated at 50 °C for 5 days. Then, the bacterial cultures were diluted with tap water (1:1 *v*/*v*) and 200 mL of the diluted cultures were poured onto the rhizosphere of each in the co-inoculation treatment group. For the non-inoculated control group and the treatment group inoculated with only *M. phaseolina* or *F. oxysporum f. sp. fragariae*, an equal volume (200 mL) of tap water was applied at the same rate with the co-inoculation treatment group. The treatments were applied at a 7-day interval, 8 times from May to June 2020. The experiment was conducted using a completely randomized block design with 3 replications of 5 plants each and a total of 90 seedlings were used.

### 2.6. Measurement of Strawberry Growth and Fruit Yield

To determine the growth characteristics, all seedlings in each treatment group were collected at the end of June 2020 and cleaned of all the media and debris. The seedlings were separated into shoots and roots and oven-dried at 80 °C for 72 h. Then, the biomass of shoots and roots was measured and recorded.

Fruits were harvested from each pot through May and June 2020, when fruits were fresh and ripe, to determine the fruit yield. The total weight of fruits per plant was recorded and summed up for the study period.

### 2.7. Statistical Analysis

Statistical analyses were performed using SPSS (Statistical Package for the Social Sciences), version 25 (Armonk, NY, USA). Plant biomass and fruit yield data were subjected to an analysis of variance (ANOVA). The mean values were compared using Fisher’s least significant difference (LSD) test at *p* = 0.01.

## 3. Results

### 3.1. Inhibitory Effect of Bacillus velezensis CE 100 against Strawberry Fungal Pathogens

#### 3.1.1. Cell Growth of Bacillus velezensis CE 100

**Figure 1:**
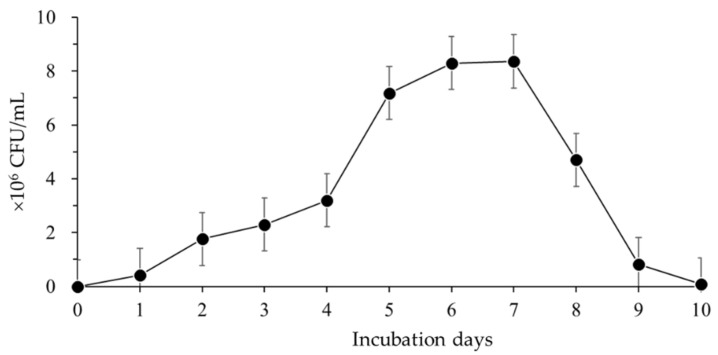
Colony-forming units (CFUs) of *Bacillus velezensis* CE 100 during the incubation period. Error bars represent standard deviation (*n* = 3).

*Bacillus velezensis* CE 100 showed a gradual increase in cell growth until four days after incubation (Figure 1). The rate of cell growth then rapidly increased from 3.2 × 10^6^ to 7.18 × 10^6^ CFU/mL between 4 and 5 days of incubation. Thereafter, the growth of *B. velezensis* CE 100 increased until 7 days after inoculation, when the growth rate reached a maximum value of 8.4 × 10^6^ CFU/mL. The growth of *B. velezensis* CE 100 sharply declined from 8 days post-inoculation until the end of the experimental period, as the bacteria depleted the nutrients supplied in the media, and cell growth was negligible from 9 days to 10 days after inoculation (Figure 1).

#### 3.1.2. Chitinase and β-1,3-Glucanase Activity of Bacillus velezensis CE 100

**Figure 2:**
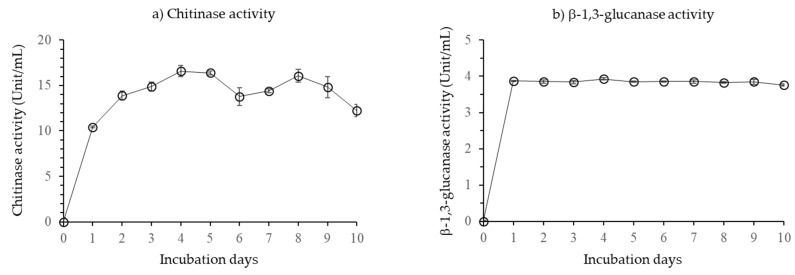
Changes in the activity of chitinase (**a**) and β-1,3-glucanase (**b**) secreted by *Bacillus velezensis* CE 100 during the incubation period. Error bars represent the standard deviation (*n* = 3).

When cultured in the liquid media supplemented with sucrose as an energy source and other essential nutrients, *B. velezensis* CE100 produced strong hydrolytic enzyme activity during growth. Specifically, the chitinase activity produced by *B. velezensis* CE100 rapidly increased in the first day after inoculation to 10.5 unit/mL, and then attained the maximum value of 16.6 unit/mL after 5 days (Figure 2a). Chitinase activity then slightly fluctuated between 5 to 8 days after inoculation, before gradually decreasing towards the end of the experiment, which is consistent with the decline in cell growth (Figure 2a).

For β-1,3-glucanase enzyme activity, *B. velezensis* CE 100 exhibited a rapid increase from zero at the start of the experiment to 3.93 unit/mL, after 1 day after incubation (Figure 2b). Then *B. velezensis* CE 100 maintained relatively constant production of β-1,3-glucanase enzyme from the first day after inoculation throughout the experiment, regardless of the changes in cell growth (Figure 2b).

#### 3.1.3. Antagonistic Activity of *Bacillus velezensis* CE 100 against Fungal Pathogens

**Figure 3:**
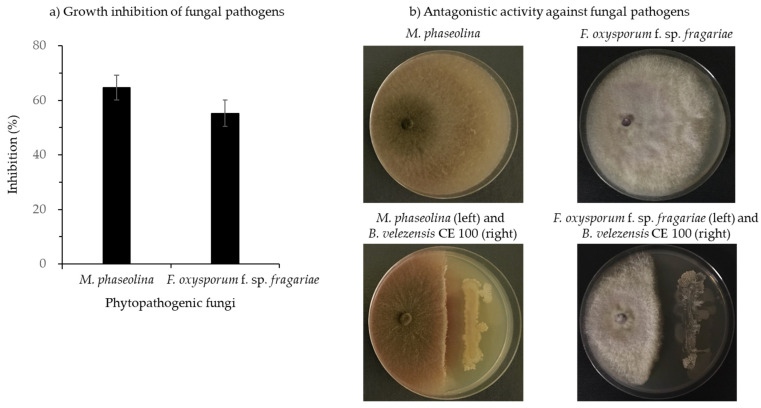
Inhibition of the mycelial growth *Macrophomina phaseolina* and *Fusarium oxysporum* f. sp. *fragariae* by *Bacillus velezensis* CE 100 (**a**) and antagonistic activity of *B. velezensis* CE 100 against *M. phaseolina* and *F. oxysporum* f. sp. *fragariae* (**b**), using the dual culture method on PDA medium. Error bars represent the standard deviation (*n* = 3).

**Figure 4:**
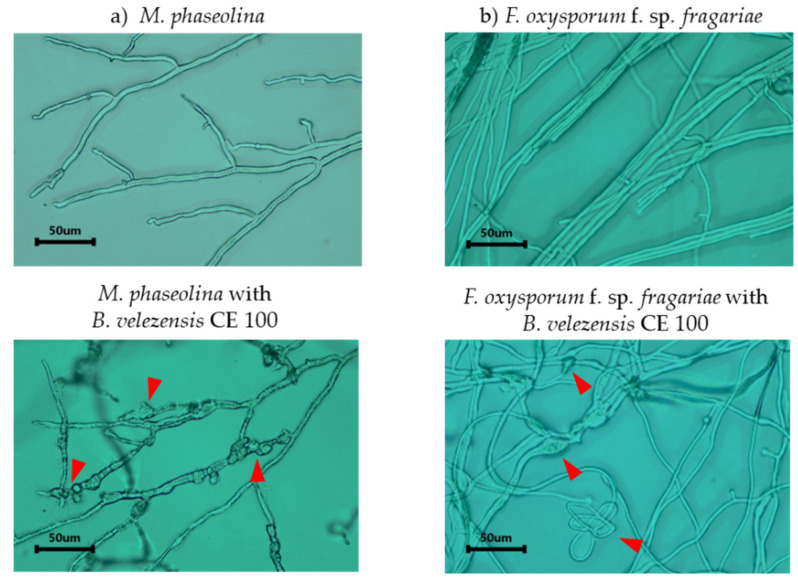
Inhibition effect of *Bacillus velezensis* CE 100 on phytopathogenic hyphal morphologies of *Macrophomina phaseolina* (**a**) and *Fusarium oxysporum* f. sp. *fragariae* (**b**), observed via light microscopy. On the top, the normal morphology in the control groups, and at the bottom, the corresponding effect of *B. velezensis* CE 100. Arrows indicate hyphal alterations with swelling and deformation of structures caused by *B. velezensis* CE 100.

*B. velezensis* CE 100 caused 64.7% and 55.2% growth inhibition against *M. phaseolina* and *F. oxysporum* f. sp. *fragariae*, respectively (Figure 3). When observed on a light microscope, the hyphal morphologies of both phytopathogenic fungi, *M. phaseolina,* and *F. oxysporum* f. sp. *fragariae* in dual culture with *B. velezensis* CE 100 showed striking differences compared to the control group, grown as a pure culture without bacterial antagonism (Figure 4). The treatment groups for both fungal pathogens showed mycelial abnormalities, such as degradation, deformation, and cell wall lysis of the hyphae compared to normal hyphae structure in the control group (Figure 4). 

**Figure 5:**
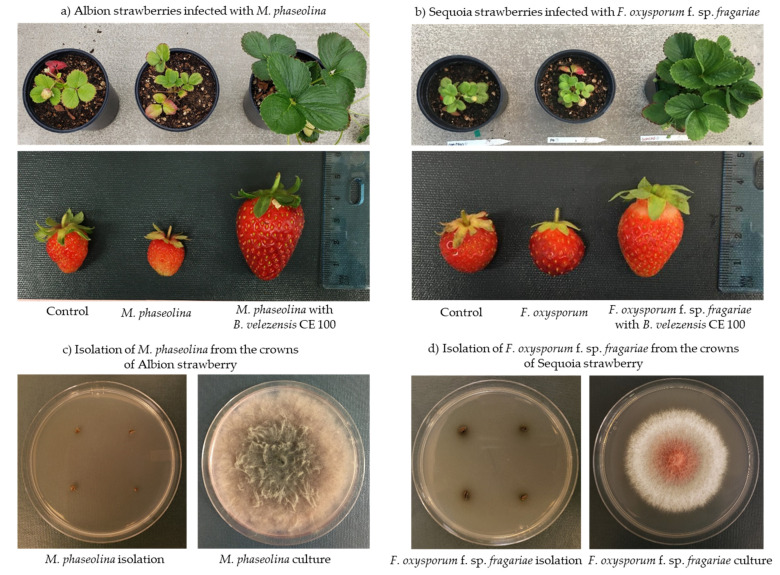
Effects of different treatments on growth and fruit production in Albion strawberries infected with *Macrophomina phaseolina* (**a**) and Sequoia strawberries inoculated with only *Fusarium oxysporum* f. sp*. fragariae* (**b**). Isolation of *M. phaseolina* (**c**) and *F. oxysporum* f. sp*. fragariae* (**d**) from infected crowns of the Albion and Sequoia strawberry plants, respectively.

In the greenhouse experiment, co-inoculation of *B. velezensis* CE 100 with *M. phaseolina* or *F. oxysporum* f. sp. *fragariae* suppressed the disease symptoms and improved the growth vigor in both Albion and Sequoia strawberry cultivars. However, strawberry plants in the non-inoculated control (without phytopathogenic fungi or bacteria) and plants inoculated with only *M. phaseolina* or *F. oxysporum f. sp. fragariae* exhibited diseases symptoms such as reddish-brown necrosis in older leaves and leaf chlorosis (turning from green to pale-green). These disease symptoms were consistent with crown-rot pathogen infections and fusarium wilt disease (Figure 5a,b). Except for the treatment groups co-inoculated with bacterial and fungal pathogens, all others plants exhibited disease symptoms such as stunted growth, leaf chlorosis, and premature leaf death. The older leaves initially turned reddish-brown along the margins followed by wilting. Thus, the spread of *M. phaseolina* and *F. oxysporum* f. sp*. fragariae* occurred throughout all the treatment groups including the non-inoculated control groups, mainly due to water splashes during irrigation. Moreover, the phytopathogenic fungi *M. phaseolina* or *F. oxysporum* f. sp. *fragariae* were successfully recovered (re-isolated) from strawberry plants in both the non-inoculated control groups and the groups inoculated with only phytopathogenic fungi. However, the recovery of both phytopathogens was not successful in the treatment groups co-inoculation of *B. velezensis* CE 100 (Figure 5c,d).

### 3.2. Production of Indole-3-Acetic Acid (IAA) by Bacillus velezensis CE 100

**Figure 6:**
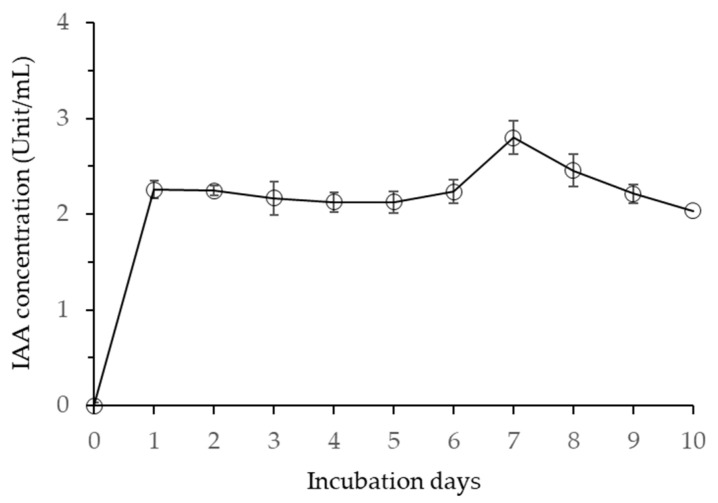
Indole-3-acetic acid (IAA) production by *Bacillus velezensis* CE 100 at 30 °C for 10 days. Error bars represent the standard deviation (*n* = 3).

The concentration of IAA secreted by *B. velezensis* CE 100 increased rapidly from 0.0 to 2.3 units/mL on the first day of incubation (Figure 6). The concentration of IAA in the media remained relatively stable for six days before showing a sharp increase to reach the maximum value of 2.8 units/mL after 7 days of incubation.

The concentration of IAA then started to decline from 2.8 unit/mL on day 7 to the lowest value of 2.0 unit/mL after 10 days of incubation (Figure 6). The highest IAA concentration in the broth culture of *B. velezensis* CE 100 at 7 days after inoculation also corresponded to the time of maximum cell growth, as observed in Figure 1. Similarly, the decline in IAA concentration from 7 days to 10 days after inoculation was also consistent with the pattern of decline in the viable cells of *B. velezensis* CE 100 (Figure 1).

### 3.3. Effect of Bacillus velezensis CE 100 on Fruit Yield

For the non-inoculated control plants and plants infected with either of the phytopathogenic fungi without bacterial treatment, the total plant dry mass was significantly lower compared to the group inoculated with *B. velezensis* CE 100 (Table 1). Moreover, the non-inoculated control plants and plants infected with either of the phytopathogenic fungi had significantly lower fruit yields compared to those receiving bacterial treatment (Table 1).

Co-inoculation with *B. velezensis* CE 100 with *M. phaseolina* or *F. oxysporum* f. sp. *fragariae* in strawberry plants resulted in a significant increase in plant dry mass compared to both the non-inoculated control groups and the groups inoculated with only phytopathogenic fungi without bacterial treatment (Table 1). Specifically, co-inoculation with *B. velezensis* CE 100 in strawberry plants infected with *M. phaseolina* or *F. oxysporum* f. sp. *fragariae* resulted in an increase in shoot dry mass by 6.2-fold or 9.2-fold, respectively. In addition, co-inoculation with *B. velezensis* CE 100 in strawberry plants infected with *M. phaseolina* or *F. oxysporum* f. sp. *fragariae* also increased shoot dry mass by 7.9-fold or 10.2-fold compared to the non-inoculated control groups.

Moreover, co-inoculation with *B. velezensis* CE 100 significantly increased the fruit yield compared to non-inoculated control plants and plants only inoculated with fungal pathogens (Table 1). The co-inoculation with *B. velezensis* CE 100 in the group infected with *M. phaseolina* or *F. oxysporum* f. sp. *fragariae* increased strawberry productivity compared to only fungal inoculation by 10.6-fold or 4.9-fold, respectively. Co-inoculation with *B. velezensis* CE 100 in plants infected with *M. phaseolina* or *F. oxysporum* f. sp. *fragariae* also improved strawberry yield compared to the non-inoculated control group, by 14.7-fold or 15.8-fold respectively.

## 4. Discussion

### 4.1. Antagonism of Macrophomina phaseolina and Fusarium oxysporum by Cell Wall-Degrading Enzymes Produced by Bacillus velezensis CE 100

*Bacillus* spp. are a versatile group of PGPB that have exhibited strong biocontrol activity against various fungal, bacterial, and viral plant pathogens [41,42,47,48,49]. Specifically, *Bacillus* spp. have been reported to control various plant fungal pathogens of economic importance in strawberry production, such as *Botrytis cinerea, F. oxysporum* f. sp. *fragariae*, and *M. phaseolina* [6,9,41]. *Bacillus* spp. control plant phytopathogenic fungi through various mechanisms, including the production of secondary metabolites such as antibiotics and hydrolytic enzymes that degrade the fungal cell wall [26,27,29,50,51,52]. Specifically, *B. velezensis* CE 100 produces antifungal biomolecules such as cyclic tetrapeptides which inhibit phytopathogenic fungal growth and sporulation [26]. However, the antifungal cyclic tetrapeptides from *B. velezensis* CE 100 are produced at very low concentrations that do not effectively cause inhibitory activity under field conditions. For instance, cyclic tetrapeptide only caused 18.8% mycelial growth inhibition of *Colletotrichum gloeosporioides* at 1000 µg/mL, and no inhibition was observed at 500 µg/mL [26]. In addition, the concentration of cyclic tetrapeptide from *B. velezensis* CE 100 culture broth was determined to be only 330 µg/mL, which is too low to cause an antifungal effect. Therefore, the biocontrol activity of *B. velezensis* CE 100 against plant phytopathogens under field conditions was demonstrated to be the effect of cell wall-degrading enzymes [27,29,50]. For instance, *B. velezensis* secretes lytic enzymes such chitinase and β-1,3-glucanase which degrade the chitin and β-glucan polymers, which are major structural components of the cell wall matrix in phytopathogenic fungi/oomycetes, leading to hyphal deformation and mycelial growth inhibition [29,32,50,52]. Some biocontrol agents have been reported to antagonize the growth of *M. phaseolina* and *F. oxysporum* f. sp. *fragariae*, and thus could potentially reduce the incidence of crown rot and fusarium wilt infections in strawberries [6,9]. In the present study, *B. velezensis* CE 100 produced high levels of chitinase and β-1,3-glucanase (Figure 2). These lytic enzymes could be involved in the antagonistic activity of *B. velezensis* CE 100 against soil-borne pathogens, such as *M. phaseolina* and *F. oxysporum* f. sp. *fragariae* (Figure 3). From the results of the dual culture assays of *B. velezensis* CE 100 with *M. phaseolina* and *F. oxysporum* f. sp. *fragariae*, the mycelia of both fungi showed severe abnormalities, such as swelling, deformation, and degradation of the hyphae, which indicates the disintegration of the cell wall matrix (Figure 4). In the greenhouse experiment, co-inoculation of *B. velezensis* CE 100 culture with *M. phaseolina* or *F. oxysporum* f. sp. *fragariae* suppressed the disease symptoms and enhanced the growth vigor of strawberry plants (Figure 5a,b). However, strawberry plants in both the non-inoculated control groups and the groups inoculated with only the phytopathogenic fungi without bacterial inoculation exhibited severe symptoms which were consistent with charcoal rot and fusarium wilt diseases. The symptoms included reddish-brown discoloration along the margins of old leaves followed by wilting and pre-mature leaf death. The infected plants also exhibited severe chlorosis with leaves turning from green to pale green, stunted growth, and reduced yield (Figure 5a,b). The occurrence of disease symptoms in the non-inoculated control groups indicated the spreading of phytopathogens from the treatment groups due to water splashes during irrigation [53]. Thus, the effects of *M. phaseolina* and *F. oxysporum* f. sp. *fragariae* infection, such as leaf discoloration, stunted growth, and yield losses, were relatively similar in both treatment groups (groups inoculated with only the phytopathogenic fungi, and the non-inoculated control groups). Leaf discoloration could be a result of impeded nutrient and mineral transport through the vascular system due to the damage caused by the phytopathogenic fungal infection [12,13]. Co-inoculation of *B. velezensis* CE 100 prevented infection by both *M. phaseolina* and *F. oxysporum* f. sp. *fragariae* in strawberry plants. This was mainly due to the role of cell wall-degrading enzymes such as chitinase and β-1,3-glucanase which antagonize the fungal cell wall activities that are vital for survival, growth, and pathogenicity of fungal pathogens [32,50,51,52]. 

### 4.2. Effects of Bacillus velezensis CE 100 on Strawberry Growth and Fruit Production

Co-inoculation with *B. velezensis* CE 100 culture not only protected the plants infected with *M*. *phaseolina* or *F. oxysporum* f. sp. *fragariae* from severe disease symptoms but also enhanced the growth and fruit yield of strawberries. The symptoms of charcoal rot and fusarium wilt disease include chlorosis, reddish-brown necrosis on leaf margins, wilting, and premature loss of leaves. These fungi attack the root cortical and vascular tissues, thereby impeding water and nutrient transport in strawberries [6,7,11]. The reduced transport of water and mineral nutrients in the plant tissues reduces the rate of photosynthesis [6,7,8]. Such symptoms could substantially lower biomass production in strawberry plants. Thus, infected plants can exhibit severe chlorosis, wilting of leaves, stunted growth, and drastic losses in yield by more than 50%, especially under conditions of water stress [7,11,12,15]. In this study, the symptoms of charcoal rot and fusarium wilt disease were prevalent in both the non-inoculated control groups and the groups inoculated with only phytopathogenic fungi without bacterial treatment (Figure 5a,b). Consequently, the non-inoculated control groups and the groups only inoculated with the phytopathogenic fungi exhibited stunted growth, low biomass, and low fruit yield due to infections (Table 1). On the other hand, co-inoculation with *B. velezensis* CE 100 suppressed the disease symptoms, which could have consequently improved the photosynthetic rate and nutrient transport through the vascular tissues, resulting in a higher growth rate and fruit yield (Table 1).

Moreover, PGPB can also produce indole-3-acetic acid (IAA), which promotes the development of root hairs, lateral roots, and adventitious roots in plants [39,40,54]. In this study, *B. velezensis* CE 100 produced IAA auxin during its growth (Figure 6). The IAA produced by *B. velezensis* CE 100 enhanced the growth and development of root hairs and lateral roots, which led to an increase in root biomass (Table 1). The enhanced root development increases the root surface area, which improves the absorption of nutrients from the soil and consequently enhances plant growth and productivity [27,29,50]. Moreover, *B. velezensis* CE 100 has been reported to produce ammonium during its growth phase [29]. Ammonium is a precursor for nitrate in the soil, and thus its release into the rhizosphere increases nitrogen availability for plant uptake [29,50]. This consequently improves the photosynthetic rate and enhances plant growth and productivity [29,40,50]. In addition, *B. velezensis* CE 100 has been reported to exhibit the potential for phosphate solubilization [29]. The solubilization of insoluble phosphate increases the availability of phosphorus in the soil and thus enhances the root architecture and nutrient absorption by plants [29,50]. The present study showed significant increases in strawberry growth and fruit production in the treatment groups co-inoculated with *B. velezensis* CE 100 (Table 1).

*M.phaseolina* and *F. oxysporum* f. sp. *fragariae* infections can cause devastating losses in strawberry production [6,7,11]. Indeed, the non-inoculated control and the treatment group inoculated with only phytopathogenic fungi exhibited extremely low biomass and fruit yield due to phytopathogenic fungal infections (Table 1). On the other hand, co-inoculation of phytopathogenic fungi with *B. velezensis* CE 100 suppressed the fungal disease symptoms through the lytic enzyme activity of chitinase and β-1,3-glucanase. Moreover, *B. velezensis* CE 100 exhibits several plant growth-promoting traits, such as IAA and ammonium production and phosphate solubilization. Therefore, *B. velezensis* CE 100 is not only an effective biocontrol agent against strawberry diseases caused by *M. phaseolina* and *F. oxysporum* f. sp. *fragariae* but also releases plant growth factors that enhance the growth and fruit yield of strawberries.

## 5. Conclusions

The findings of this study demonstrate the antagonistic effect of *B. velezensis* CE 100 against plant phytopathogenic fungi and its potential to enhance strawberry production. *B. velezensis* CE 100 produced chitinase and β-1,3-glucanase enzymes, which degraded the fungal cell walls and effectively inhibited mycelial growth of *M. phaseolina* and *F. oxysporum* f. sp. *fragariae* fungi, the causal agents of charcoal rot and fusarium wilt diseases in strawberry, respectively. Co-inoculation of *B. velezensis* CE 100 with the phytopathogenic fungi effectively suppressed the symptoms associated with charcoal rot and fusarium wilt diseases in strawberries. In addition, *B. velezensis* CE 100 produced IAA, which enhances root hair growth and lateral root development. The production of IAA not only improves plant nutrient uptake, but also facilitates cell division and differentiation. Our results confirmed that inoculation of *B. velezensis* CE 100 leads to increased plant growth, biomass production, and fruit yield in strawberries. Therefore, this study demonstrates the potential of *B. velezensis* CE 100 as a biocontrol alternative to chemical fungicides in the management of fungal diseases caused by *M. phaseolina* and *F. oxysporum* f. sp. *fragariae*, and a bio-stimulant to enhance strawberry growth and fruit yield.

## Figures and Tables

**Table 1:** Effects of different treatments on plant biomass and fruit yield in strawberry plants after inoculation with *Macrophomina phaseolina or Fusarium oxysporum* f. sp. *fragariae*.

Treatments	Plant Biomass (g/Plant)			Fruit Yield (g/Plant)
	Shoot	Root	Total	
Control	1.1 ± 0.2 ^b^	1.2 ± 0.5 ^b^	2.4 ± 0.6 ^b^	1.6 ± 1.9 ^b^
*M. phaseolina*	1.4 ± 0.5 ^b^	1.5 ± 0.6 ^b^	2.9 ± 1.1 ^b^	2.2 ± 4.8 ^b^
*M. phaseolina* with *B. velezensis* CE 100	8.7 ± 2.7 ^a^	2.1 ± 0.5 ^a^	10.8 ± 3.2 ^a^	32.3 ± 13.5 ^a^
Control	1.0 ± 0.3 ^b^	1.1 ± 0.4 ^b^	2.1 ± 0.6 ^b^	1.6 ± 2.0 ^b^
*F. oxysporum* f. sp. *fragariae*	1.1 ± 0.4 ^b^	1.4 ± 0.5 ^b^	2.5 ± 0.6 ^b^	5.2 ± 3.2 ^b^
*F. oxysporum* f. sp. *fragariae* with *B. velezensis* CE 100	10.2 ± 3.9 ^a^	1.9 ± 0.4 ^a^	12.2 ± 4.0 ^a^	25.3 ± 7.7 ^a^

Means with different superscripts a and b in the same column indicate significantly different values, at *p* = 0.01 when compared using the LSD test.

## Data Availability

All the data relevant to this manuscript is available on request from the corresponding author.
